# Peripheral Airway Dysfunction Assessed by Impulse Oscillometry in School-Aged Children Born Preterm: A Cross-Sectional Comparative Study

**DOI:** 10.3390/jcm15176692

**Published:** 2026-08-28

**Authors:** Nezihe Koker Ozer, Ercan Yılmaz, Hatice Turgut, Erdem Topal, Ramazan Ozdemir, Recep Gunakın, Mehmet Aslan

**Affiliations:** 1 Department of Pediatrics, Faculty of Medicine, Inonu University, Malatya 44280, Turkeyrecep.gunakin@inonu.edu.tr (R.G.);; 2 Department of Pediatric Allergy and Immunology, Faculty of Medicine, Inonu University, Malatya 44280, Turkey; ercanyilmz44@gmail.com; 3 Department of Neonatology, Faculty of Medicine, Inonu University, Malatya 44280, Turkey

**Keywords:** impulse oscillometry, peripheral airways, prematurity

## Abstract

**Background:** Preterm birth may result in persistent alterations in peripheral airway development. We aimed to compare impulse oscillometry (IOS) parameters between school-aged children born preterm and healthy term-born controls and to investigate factors associated with peripheral airway dysfunction. **Methods:** This cross-sectional comparative study included 46 children born at ≤32 weeks of gestation and 40 independently recruited healthy term-born controls. IOS parameters, including R5, R20, R5–R20, X5, X20, AX, and Fres, were assessed. Multivariable linear regression was used to evaluate whether between-group differences persisted after adjustment for anthropometric measures. **Results:** Children born preterm had significantly higher R5 (0.75 vs. 0.48 kPa·s/L), R5–R20 (0.37 vs. 0.19 kPa·s/L), and AX (2.93 vs. 1.41 kPa/L) than controls (all *p* = 0.001), whereas R20, X5, X20, and Fres did not differ significantly. R5–R20 was the most frequently abnormal IOS parameter in the preterm group (34.8%). After adjustment for age, height z-score, and weight z-score, prematurity remained significantly associated with higher R5 (*p* = 0.005), R5–R20 (*p* < 0.001), and AX (*p* = 0.002). No significant associations were identified between R5–R20 and gestational age, bronchopulmonary dysplasia, mechanical ventilation, or other evaluated neonatal characteristics. **Conclusions:** School-aged children born preterm exhibit persistent differences in peripheral airway mechanics that are not explained by anthropometric differences. IOS may provide a useful complementary tool for long-term respiratory assessment after preterm birth.

## 1. Introduction

Although neonatal care has improved considerably, preterm birth continues to be an important contributor to long-term morbidity during childhood. With the marked increase in survival rates in recent years, research has shifted its focus not only to complications in the early stages of life but also to long-term health outcomes extending into school-age and adulthood. In particular, as a large proportion of lung development continues after birth, it is thought that airway development in preterm infants may be permanently affected. This situation is not limited to patients who develop bronchopulmonary dysplasia (BPD); long-term respiratory dysfunction has been reported even in children who experienced no significant respiratory complications during the neonatal period [1,2,3].

Disruption of alveolarization and the development of the peripheral airways in individuals born prematurely may set the stage for peripheral airway dysfunction later in life. Structural changes occurring in early life may, over time, result in airway remodeling, loss of elastic recoil and an increase in peripheral airway resistance. Although these changes may remain clinically asymptomatic, they have been associated with long-term functional impairment and, in some individuals, respiratory phenotypes resembling chronic obstructive pulmonary disease in adulthood. Consequently, the assessment of the peripheral airways in preterm infants is becoming increasingly important [4,5,6].

Spirometry is the standard method for assessing airflow limitation and provides an integrated measure of respiratory function; however, conventional spirometric indices may be relatively insensitive to subtle abnormalities involving the peripheral airways. Impulse oscillometry (IOS) is a noninvasive pulmonary function technique that applies small pressure oscillations to the respiratory system during quiet tidal breathing and measures respiratory resistance and reactance across different frequencies, providing complementary information on central and peripheral airway mechanics. Because IOS requires minimal patient cooperation, it is particularly useful in children who may have difficulty performing technically acceptable forced expiratory maneuvers. Thus, IOS complements rather than replaces spirometry and may provide additional insight into subtle peripheral airway abnormalities [7,8,9].

Studies using IOS in preterm infants, however, remain limited, and most existing research has focused on cases developing BPD or on early childhood. Furthermore, there are very few studies that have determined the prevalence of peripheral airway dysfunction in school-aged preterm children and assessed the rates of abnormality in IOS parameters relative to normal reference z-scores. In addition, the existing literature contains conflicting results regarding the association between neonatal risk factors and long-term IOS findings [10,11,12,13].

Therefore, the aim of this study was to compare peripheral airway function in school-aged preterm children with that of healthy peers using impulse oscillometry, to determine the prevalence of abnormalities in IOS parameters, and to investigate the possible effects of perinatal and neonatal risk factors on long-term peripheral airway function.

## 2. Materials and Methods

### 2.1. Study Design and Study Population

This was a single-center, cross-sectional comparative study conducted to assess respiratory mechanics in school-aged children born prematurely using impulse oscillometry (IOS). Eligible children born preterm were identified from hospital records before the assessment period and were subsequently contacted and invited to participate. Clinical assessments and IOS measurements were conducted during a predefined testing period between 29 June and 15 July 2026.

The study group comprised children who had been monitored in the Neonatal Intensive Care Unit of the Faculty of Medicine at Inonu University during the neonatal period, had a gestational age of ≤32 weeks, and were aged 7–8 years at the time of the study. Following approval from the ethics committee, potentially eligible patients were identified using the neonatal intensive care unit’s electronic record system. Parents were contacted by telephone using the contact details on file, and those who agreed to participate were invited to the Pediatric Allergy Outpatient Clinic for assessment. All assessments were completed during a single outpatient clinic visit on the same day.

The preterm group included children born at ≤32 weeks of gestation who were aged 7–8 years at the time of assessment and had complete neonatal clinical data available. Participants were also required to complete a technically acceptable IOS measurement in accordance with ERS criteria [7] and to have written informed consent provided by a parent or legal guardian. Healthy term-born children aged 7–10 years were recruited as controls from children attending the outpatient clinics for routine visits and from children of hospital staff. Potential controls were evaluated according to the predefined inclusion and exclusion criteria before study enrollment. The exact number of potentially eligible control children initially screened was not systematically recorded and was therefore unavailable from the recruitment records. The control group consisted of healthy term-born children aged 7–10 years without chronic disease. Controls were recruited independently of the preterm group, and no individual or frequency matching procedure was applied. Of the 45 eligible control children who underwent study assessment, five were excluded because they were unable or unwilling to complete IOS testing, resulting in 40 controls being included in the final analysis.

For both groups, congenital heart disease, cystic fibrosis, primary ciliary dyskinesia, primary immunodeficiency, interstitial lung disease, chest wall deformity, neuromuscular disease, severe neurodevelopmental disorders that could affect the reliability of the measurements, and upper or lower respiratory tract infections experienced within the previous four weeks were identified as exclusion criteria.

**Figure 1:**
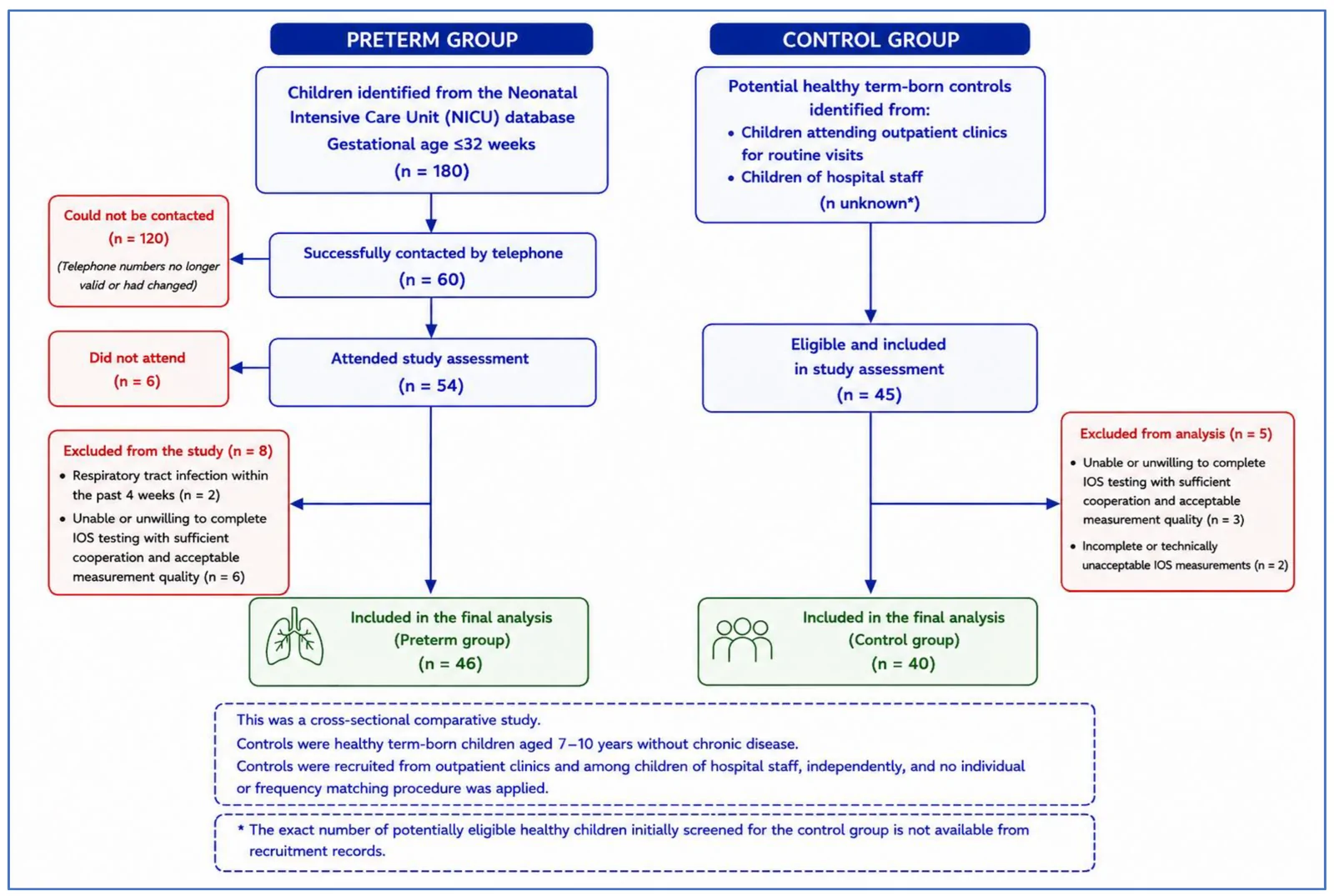
Study Flow Diagram.

A total of 180 children with a gestational age of ≤32 weeks were identified from neonatal intensive care unit records. Among the children successfully contacted by telephone, 54 agreed to attend the outpatient clinic for further assessment. Of the 54 children assessed, 8 were excluded from the study; 2 of these were excluded due to having had an upper respiratory tract infection within the previous four weeks at the time of assessment, whilst 6 were excluded because they were unable to complete a technically acceptable impulse oscillometry measurement or did not meet the other inclusion criteria. Consequently, 46 children from the preterm group were included in the analyses. The recruitment, assessment, and inclusion of participants in both the preterm and control groups are summarized in the study flow diagram (Figure 1).

### 2.2. Collection of Perinatal and Neonatal Data

Perinatal and neonatal data on preterm infants were obtained from hospital electronic records, neonatal intensive care unit files and interviews with parents. The variables recorded included gestational age, birth weight, sex, small for gestational age, administration of prenatal corticosteroids, chorioamnionitis, surfactant therapy, the need for and duration of mechanical ventilation, the duration of continuous positive airway pressure therapy, the duration of oxygen therapy, length of stay in the neonatal intensive care unit, neonatal sepsis, neonatal apnea, bronchopulmonary dysplasia, necrotizing enterocolitis, patent ductus arteriosus and intraventricular hemorrhage.

Small for gestational age was defined as a birth weight below the 10th percentile for gestational age and sex. The diagnosis of bronchopulmonary dysplasia was determined from neonatal records based on the continued need for oxygen or respiratory support at corrected 36 weeks’ gestation or at the time of discharge.

### 2.3. Clinical Assessment

The demographic characteristics and current clinical histories of all children were recorded on a standard data form following face-to-face interviews with their parents. Age, gender, consanguinity between parents, duration of breastfeeding, exposure to environmental tobacco smoke and the presence of pets were recorded.

In the respiratory assessment, medically diagnosed asthma, recurrent wheezing, hospitalization due to lower respiratory tract infection, long-term use of inhaled corticosteroids and a family history of asthma were recorded. With regard to atopic diseases, allergic rhinitis, atopic eczema, food allergy and a family history of atopic diseases were assessed.

### 2.4. Laboratory Assessment

In the preterm group, peripheral blood eosinophil counts and total immunoglobulin E levels were recorded from patient records or from laboratory results obtained during the study assessment. Peripheral eosinophil counts were expressed as ×10^3^/mm^3^, and total IgE levels as IU/mL.

Sensitivity to aeroallergens was assessed based on skin prick test results and/or serum-specific IgE levels. A positive skin prick test to at least one aeroallergen or a serum-specific IgE level of ≥0.35 kUA/L was considered indicative of sensitivity to aeroallergens. The aeroallergens assessed, in accordance with the center’s routine panel, included house dust mites, grass and tree pollens, weed pollens, molds, and cat dander.

### 2.5. Impulse Oscillometry Measurement and Quality Control

Airway resistance and reactance were assessed using an impulse oscillometry system (Vyntus™ IOS, Vyaire Medical GmbH, Höchberg, Germany). Before each testing session, calibration of the IOS device was performed according to the manufacturer’s recommendations using a standard 3-L syringe and reference resistance.

IOS testing was conducted according to the technical recommendations for respiratory oscillometry issued by the European Respiratory Society (ERS) and endorsed by the American Thoracic Society (ATS) [7]. Participants were instructed to avoid heavy meals and vigorous exercise before testing. In participants using short-acting bronchodilators, these medications were withheld for at least 4–6 h prior to IOS assessment in accordance with the testing protocol. All children were clinically stable on the day of testing, and those with a recent respiratory tract infection were assessed only after meeting the predefined eligibility criteria. The assessor performing the IOS measurements was not blinded to participants’ birth status or neonatal history. IOS testing was carried out in a quiet setting while participants were seated with the head in a neutral position and a nose clip in place. During the measurements, participants supported their cheeks and the floor of the mouth with their hands to reduce upper airway artifacts.

For each IOS recording, participants breathed quietly through the device mouthpiece for approximately 30–45 s while maintaining an adequate seal. A minimum of three recordings meeting the predefined technical acceptability criteria were obtained for each participant. Reproducibility of IOS recordings was evaluated based on the coefficient of variation (CV) for R5, with a CV below 15% required for inclusion in the analysis. Recordings were accepted only when tidal breathing remained stable and no technical artifacts, including coughing, swallowing, vocalization, air leakage, or irregular breathing, were present. In addition, coherence values were required to be ≥0.80 at 5 Hz and ≥0.90 at frequencies ≥10 Hz. For each participant, the final IOS values were calculated as the mean of the technically acceptable recordings.

### 2.6. Reference Equations and Definition of Abnormal IOS Parameters

Airway resistance was characterized using R5, representing total respiratory resistance at 5 Hz; R20, representing resistance at 20 Hz and predominantly reflecting the more central airways; and R5–R20, representing frequency dependence of resistance and used as an index of peripheral airway involvement. Reactance was assessed using X5 and X20, together with the area of reactance (AX) and resonance frequency (Fres). More negative reactance values were interpreted as indicating greater elastic loading and peripheral airway involvement.

Reference equations were specified separately for each IOS parameter. For R5, R20, X5, X20, and Fres, predicted values and z-scores were obtained from the Dencker/Malmberg (2006) [14] reference set incorporated into the software. The original reference equations of Dencker et al. were derived from 360 healthy children aged 2.1–11.1 years, recruited from kindergartens in Finland and primary schools in Sweden, with a height range of approximately 90–160 cm. IOS measurements in that reference population were performed using a Jaeger IOS system. Respiratory resistance and reactance were measured at 5, 10, 15, and 20 Hz, together with total respiratory impedance and resonance frequency. All oscillometric variables were significantly related to height, while most showed an additional weaker relationship with body weight; the parameter-specific regression equations implemented in the software were used to generate predicted values and corresponding z-scores [14].

Because R5–R20 and AX were not accompanied by corresponding z-scores in the Dencker/Malmberg reference module available in our SentrySuite configuration, z-scores for these two parameters were calculated separately using the equations published by Gochicoa-Rangel et al. (2023) [15]. These reference equations were derived from 830 healthy nonsmoking subjects aged 2.7–90 years, 54.0% of whom were female and 54.8% of whom were children aged ≤12 years; most participants resided in Mexico City, Mexico. The authors used multivariable segmented regression models including sex, age, height, and body mass index (BMI) as independent variables and provided parameter-specific coefficients for calculation of predicted values, lower and upper limits of normal, percentage predicted values, and z-scores [15]. For R5–R20 and AX, calculations were performed using the publicly accessible IOS Z-score Calculator “https://ioszscore.manus.space/ (accessed on 20 August 2026)”, which was used to apply the published Gochicoa-Rangel equations. The required demographic and anthropometric variables and the observed IOS value were entered for each participant. The calculator output was verified against the coefficients and equations reported in the original publication, with no modifications to the published methodology. For both reference sets, abnormal values were defined using the same z-score thresholds: z > +1.645 for R5, R20, R5–R20, AX, and Fres, and z < −1.645 for X5 and X20. The prevalence of abnormal findings was then calculated separately for each IOS parameter.

### 2.7. Sample Size and Power Considerations

R5–R20 was considered the principal IOS outcome for interpretation because it reflects frequency-dependent peripheral airway resistance. No a priori sample-size calculation was performed because recruitment was constrained by the number of eligible children born very preterm who could be reassessed at school age. A sample-size sensitivity analysis based on the final sample (46 preterm and 40 control participants) indicated that, with a two-sided α of 0.05 and 80% power, the study could detect a standardized between-group difference of approximately Cohen’s d = 0.61 or greater. Thus, the available sample was primarily capable of detecting moderate-to-large between-group effects, whereas smaller effects, particularly in secondary and subgroup analyses, may have remained undetected. Secondary IOS, neonatal, and clinical analyses were considered exploratory; no adjustment for multiple comparisons was applied, and these findings were interpreted cautiously.

### 2.8. Use of Generative Artificial Intelligence

Generative artificial intelligence (AI) tools were used solely to assist with English-language and grammatical editing of the manuscript and with the preparation of Figure 1. AI tools were not used for study conception or design, data collection or generation, statistical analysis, interpretation of the results, or development of the scientific discussion or conclusions. All scientific content and AI-assisted outputs were critically reviewed and verified by the authors, who take full responsibility for the final content.

### 2.9. Statistical Analysis

Statistical analyses were performed using IBM SPSS Statistics (Version 21.0; IBM Corp., Armonk, NY, USA). The distribution of continuous variables was assessed using the Kolmogorov–Smirnov test. Categorical variables were expressed as frequencies and percentages, whereas non-normally distributed continuous variables were expressed as median (Q1-Q3). Between-group comparisons were performed using Pearson’s chi-square test or Fisher’s exact test for categorical variables and the Mann–Whitney U test for continuous variables, as appropriate. Associations between oscillometric parameters and continuous demographic, perinatal, neonatal, and laboratory variables were evaluated using Spearman’s rank correlation coefficient.

Given the exploratory nature of the analyses of neonatal and clinical predictors, no adjustment for multiple comparisons was applied; these analyses were considered hypothesis-generating and interpreted cautiously. To account for potential confounding by anthropometric differences between the groups, multivariable linear regression analyses were performed for IOS parameters that differed significantly between the preterm and control groups (R5, R5–R20, and AX). In these models, study group (preterm vs. healthy control) was entered as the main independent variable, with age, height z-score, and weight z-score included as covariates. Finally, an analysis restricted to the preterm cohort was performed to evaluate associations of gestational age, BPD, and mechanical ventilation with R5–R20. Regression results were reported as unstandardized coefficients (B), 95% confidence intervals (CIs), standardized coefficients (β), and *p* values. All statistical tests were two-tailed, and a *p* value < 0.05 was considered statistically significant.

## 3. Results

### 3.1. Baseline Demographic, Neonatal, Clinical, and Laboratory Characteristics of the Preterm Cohort

A total of 46 children born preterm were included in the study. The median age at assessment was 7 years (Q1-Q3, 7.0–7.5 years), and 21 (45.7%) participants were male. The median gestational age was 29 weeks (Q1-Q3, 28–32 weeks), while 11 (23.9%) children were born small for gestational age. A history of consanguineous marriage was present in 15 (32.6%) participants.

Regarding neonatal characteristics, 30 (65.2%) children had neonatal sepsis, 20 (43.5%) experienced neonatal apnea, 16 (34.8%) had bronchopulmonary dysplasia (BPD), 19 (41.3%) required mechanical ventilation, and 40 (87.0%) received continuous positive airway pressure (CPAP). The median duration of mechanical ventilation was 1 day (Q1-Q3, 0–1 days), the median duration of oxygen therapy was 3.5 days (Q1-Q3, 1–19 days), and the median neonatal intensive care unit stay was 43 days (Q1-Q3, 19–75 days). Surfactant therapy was administered to 10 (21.7%) children, prenatal corticosteroids to 14 (30.4%), and postnatal corticosteroids to 8 (17.4%).

A history of asthma or recurrent wheezing was present in 10 (21.7%) participants, allergic rhinitis in 7 (15.2%), eczema in 3 (6.5%), and food allergy in 1 (2.2%). Family histories of asthma, allergic rhinitis, and eczema were reported in 13.0%, 23.9%, and 13.0% of participants, respectively. Motor developmental problems were identified in 11 (23.9%) children, visual impairment in 14 (30.4%), hearing impairment in 3 (6.5%), special education requirements in 6 (13.0%), and previous physical therapy in 8 (17.4%). The median total IgE level was 56 IU/mL (Q1-Q3, 15.3–148 IU/mL), the median eosinophil count was 0.29 × 10^3^/mm^3^ (Q1-Q3, 0.1–0.84 × 10^3^/mm^3^), and 12 (26.0%) participants demonstrated aeroallergen sensitization. The demographic, neonatal, clinical, and laboratory characteristics of the study participants are summarized in Table 1.

### 3.2. Comparison of Oscillometric Measurements Between the Preterm and Healthy Control Groups

The oscillometric measurements are summarized in Table 2. Demographic characteristics were comparable between the preterm and healthy control groups, with no significant differences in sex distribution, age, or smoking exposure. Among oscillometric parameters, children born preterm had significantly higher R5 (0.75 vs. 0.48 kPa·s/L, *p* = 0.001), R5–R20 (0.37 vs. 0.19 kPa·s/L, *p* = 0.001), and AX (2.93 vs. 1.41 kPa/L, *p* = 0.001) values than healthy controls, whereas R20, X5, X20, and Fres were comparable between the groups (all *p* > 0.05). These findings suggest impairment predominantly affecting the peripheral airways in children born preterm.

### 3.3. Abnormal Impulse Oscillometry Findings in the Preterm Group

The distribution of impulse oscillometry parameters and the frequency of abnormal IOS findings in the preterm group are presented in Table 3. Based on the predefined z-score criteria, R5–R20 was the most frequently abnormal parameter, with 16 children (34.8%) exceeding the upper limit of normal. Abnormal R5 values were observed in 12 (26.1%) participants, followed by abnormal AX values in 10 (21.7%). Abnormal R20, Fres, and X20 values were each observed in 9 (19.6%) participants. Reduced X5 values below the lower limit of normal were identified in 8 (17.4%) participants.

To identify factors associated with abnormal peripheral airway function, demographic, perinatal, neonatal, clinical, laboratory, environmental, and neurodevelopmental characteristics were compared between children with normal and abnormal R5–R20 values (z-score > 1.645). Chi-square analyses showed no significant associations between abnormal R5–R20 and categorical variables, including sex, prenatal or postnatal steroid exposure, chorioamnionitis, breastfeeding duration, small-for-gestational-age status, mechanical ventilation, CPAP use, surfactant administration, bronchopulmonary dysplasia, necrotizing enterocolitis, patent ductus arteriosus, intraventricular hemorrhage, neonatal apnea, physician-diagnosed asthma, recurrent wheezing, eczema, hospitalization for lower respiratory tract infection, prolonged inhaled corticosteroid use, environmental tobacco smoke exposure, pet ownership, aeroallergen sensitization, motor developmental delay, physical therapy, rehabilitation center assessment, or special education requirement (all *p* > 0.05). Likewise, Mann–Whitney U analyses demonstrated no significant differences in gestational age, neonatal intensive care unit stay, duration of mechanical ventilation, duration of oxygen therapy, peripheral eosinophil count, or total IgE levels between children with normal and abnormal R5–R20 values (all *p* > 0.05).

### 3.4. Multivariable Regression and Sensitivity Analyses

Multivariable linear regression analyses were performed to determine whether the between-group differences in R5, R5–R20, and AX persisted after adjustment for age, height z-score, and weight z-score (Table 4). Prematurity status remained associated with higher R5 (B = 0.149, 95% CI 0.047–0.252; β = 0.329; *p* = 0.005), R5–R20 (B = 0.181, 95% CI 0.101–0.260; β = 0.474; *p* < 0.001), and AX (B = 1.007, 95% CI 0.393–1.621; β = 0.370; *p* = 0.002). Age, height z-score, and weight z-score were not associated with any of the three IOS outcomes (all *p* > 0.05). The models explained 12.2%, 25.1%, and 12.5% of the variance in R5, R5–R20, and AX, respectively.

Regression diagnostics showed no substantial evidence of non-linearity, heteroscedasticity, or problematic multicollinearity. Although extreme standardized residuals were identified in the R5–R20 and AX models, sensitivity analyses excluding these observations produced materially unchanged estimates for prematurity status: R5–R20, B = 0.177 (95% CI 0.109–0.244; β = 0.538; *p* < 0.001), and AX, B = 0.999 (95% CI 0.454–1.543; β = 0.399; *p* < 0.001), supporting the robustness of the primary findings.

In a sensitivity analysis excluding the 10 preterm participants with asthma and/or recurrent wheezing, the associations between prematurity status and R5 (B = 0.136, 95% CI 0.024–0.248; β = 0.300; *p* = 0.018), R5–R20 (B = 0.181, 95% CI 0.093–0.269; β = 0.469; *p* < 0.001), and AX (B = 1.014, 95% CI 0.341–1.687; β = 0.369; *p* = 0.004) remained significant after adjustment for age, height z-score, and weight z-score.

### 3.5. Factors Associated with R5–R20 Among Children Born Preterm

Among children born preterm, univariable linear regression analyses were performed to examine the associations of individual perinatal, neonatal, post-neonatal clinical, allergic, and environmental characteristics with R5–R20. None of the evaluated characteristics showed a statistically significant association with R5–R20. For example, gestational age (B = 0.014 per week, 95% CI −0.012 to 0.040; *p* = 0.288), mechanical ventilation (B = 0.057, 95% CI −0.067 to 0.181; *p* = 0.357), and BPD (B = −0.076, 95% CI −0.208 to 0.056; *p* = 0.251) were not statistically significantly associated with R5–R20. Group summaries and effect estimate with 95% confidence intervals for all individual analyses are presented in Supplementary Table S1. In an exploratory multivariable model including gestational age, mechanical ventilation, and BPD, the overall model was not statistically significant (R^2^ = 0.036, adjusted R^2^ = −0.033; F [3,42] = 0.517; *p* = 0.673). No statistically significant associations were detected for gestational age (B = 0.006, 95% CI −0.032 to 0.044; *p* = 0.765), mechanical ventilation (B = 0.017, 95% CI −0.147 to 0.182; *p* = 0.832), or BPD (B = −0.049, 95% CI −0.223 to 0.125; *p* = 0.571).

### 3.6. Associations Between R5–R20 and Clinical, Perinatal, and Neonatal Factors

Spearman’s rank correlation analysis was performed to evaluate the relationships between R5–R20 and perinatal, neonatal, and laboratory characteristics. No significant correlations were identified between R5–R20 and gestational age (ρ = 0.099, *p* = 0.512), birth weight (ρ = −0.022, *p* = 0.884), duration of neonatal intensive care unit stay (ρ = 0.023, *p* = 0.878), duration of mechanical ventilation (ρ = −0.122, *p* = 0.417), duration of CPAP therapy (ρ = −0.086, *p* = 0.569), duration of oxygen therapy (ρ = −0.076, *p* = 0.618), total IgE levels (ρ = −0.084, *p* = 0.703), or peripheral eosinophil counts (ρ = −0.035, *p* = 0.861).

## 4. Discussion

The main finding of this study was that school-aged children born preterm exhibited differences in oscillometric measures of peripheral airway function compared with healthy term-born controls. Whilst R5, R5–R20 and AX values were found to be significantly higher in the preterm group, no significant differences were observed between the two groups in terms of R20, X5, X20 and Fres. Furthermore, R5–R20 was identified as the most frequently abnormal IOS parameter; however, no significant association was found between peripheral airway function and gestational age, birth weight, bronchopulmonary dysplasia (BPD), mechanical ventilation, duration of oxygen therapy or other neonatal variables. These findings are consistent with differences in peripheral airway mechanics at school age in children born preterm, although no clear associations with the neonatal characteristics evaluated in this study were detected.

Preterm birth occurs during one of the most critical periods of lung development. A significant proportion of alveolarization and the development of the distal airways continues during the final trimester of pregnancy and in the first few years after birth. Premature birth interrupts this physiological process, potentially resulting in a reduction in alveolar number, a decrease in airway diameter, disruption of elastic fiber organization, and inadequate pulmonary microvascular development. It is now recognized that these changes are not confined to the neonatal period but persist into later stages of life, resulting in a permanent lung phenotype defined as ‘developmental lung disease’. Consequently, prematurity is no longer regarded merely as a consequence of early birth but is considered a significant lifelong risk factor for chronic respiratory diseases in childhood and adulthood [3,4,16].

The significantly elevated R5 and, particularly, R5–R20 values suggest that the observed functional alterations in children born preterm predominantly involve the peripheral airways. Whilst R5 reflects total airway resistance, R20 assesses the central airways to a greater extent. In contrast, R5–R20 is regarded as one of the most sensitive indicators of peripheral airway involvement, as it reflects the frequency-dependent difference in resistance. The elevation of R5 and R5–R20 in the absence of a significant difference in R20 suggests that the observed differences associated with preterm birth predominantly involve the distal rather than the large airways. This finding supports the notion that peripheral airway disease may not be detectable by spirometry and that IOS may be a more sensitive assessment method in this patient group [7,8].

The clinical significance of the observed IOS differences also warrants consideration. The median R5–R20 was approximately twofold higher in the preterm group than in term-born controls (0.37 vs. 0.19 kPa·s/L), and 34.8% of children born preterm had an R5–R20 value outside the reference range. Although a validated minimal clinically important difference for R5–R20 has not been established specifically for children born preterm, the concordance between the group-level difference and the proportion of children with abnormal values supports the physiological relevance of the finding. Previous studies have demonstrated abnormal airway resistance in children born preterm, and longitudinal pediatric data outside the prematurity setting suggest that increased oscillometric resistance may identify children at increased risk of subsequent respiratory symptoms, asthma, and impaired lung function [2,10,12]. However, whether elevated R5–R20 specifically predicts future respiratory morbidity after preterm birth remains uncertain and cannot be determined from our cross-sectional study. From a clinical perspective, IOS may therefore be most useful as a complementary component of longitudinal respiratory assessment rather than as a stand-alone diagnostic or treatment-guiding test. Serial IOS assessments, particularly in children with recurrent wheezing, chronic respiratory symptoms, exercise limitation, or difficulty performing reliable spirometry, may help identify persistent or evolving peripheral airway dysfunction and prompt more detailed respiratory evaluation. Prospective longitudinal studies are needed to determine whether these IOS abnormalities predict subsequent respiratory morbidity and to define clinically meaningful thresholds in children born preterm.

One of the notable findings of our study is that the height and weight z-scores of the preterm group were significantly lower than those of the control group. This result is consistent with previous studies reporting growth retardation in preterm infants that may persist throughout childhood. Early postnatal growth restriction associated with prematurity, neonatal morbidities, feeding difficulties and changes in body composition may lead to lower height and weight values during school age [3,4,16]. Furthermore, this finding is of particular importance when interpreting IOS results. Airway resistance decreases physiologically with somatic growth, particularly with increasing height, because of the associated increase in airway caliber. Accordingly, the significantly lower height and weight z-scores in the preterm group could potentially contribute to higher resistance values. However, the between-group differences in R5, R5–R20, and AX persisted after statistical adjustment for both anthropometric measures, suggesting that differences in body size alone do not account for the observed alterations in respiratory mechanics. These findings support the presence of peripheral airway mechanical alterations beyond those attributable to somatic growth and are consistent with evidence that preterm birth may be associated with lasting alterations in distal airway development and respiratory mechanics [8,17].

Studies evaluating IOS in children born preterm remain limited. However, long-term studies based on spirometry consistently demonstrate persistent airway obstruction in childhood and adulthood. Vrijlandt et al. [10] reported airflow limitation persisting into young adulthood in individuals with very low birth weight, whilst Fawke et al. [11] demonstrated significant loss of lung function in extremely preterm children aged 11 years. Similarly, Doyle et al. [1] demonstrated that expiratory airflow in preterm-born individuals remained lower than in healthy controls even in late adolescence. In recent years, Simpson and colleagues [2] have also reported that lung function in children born prematurely follows a different developmental trajectory throughout childhood compared to their normal peers. Our findings complement these studies by showing that differences in respiratory mechanics at school age can also be detected using IOS.

It is also noteworthy that, in our study, R5–R20 was the IOS parameter most frequently found to be abnormal. Studies conducted in children with asthma have reported that R5–R20 is more sensitive than spirometry in detecting peripheral airway disease. Studies by Shi and Komarow have particularly highlighted the diagnostic value of R5–R20 in early peripheral airway obstruction. Our results suggest that this parameter may be an important biomarker not only for asthma but also for the assessment of chronic lung sequelae associated with preterm birth. The finding that R5–R20 was abnormal in approximately one-third of the preterm group indicates that peripheral airway abnormalities may be detectable even in children without overt respiratory symptoms [7,17].

Another important finding was that AX values were also found to be significantly higher in the preterm group. AX is the integral of low-frequency reactance and reflects elastic loading and ventilation heterogeneity in the distal airways. In contrast, the fact that X5 and Fres values did not differ between the two groups suggests that peripheral airway disease in the children in our study is characterized more by changes in resistance. This is consistent with the pediatric IOS literature, which indicates that R5–R20 may be more sensitive than reactance parameters in the early stages [18].

One of the unexpected findings of our study was the absence of significant associations between peripheral airway function and neonatal variables, including gestational age, birth weight, bronchopulmonary dysplasia (BPD), mechanical ventilation, and oxygen therapy. This finding may initially appear inconsistent with previous studies showing that greater prematurity and more severe neonatal respiratory disease, particularly BPD, are associated with poorer lung function at school age. For example, the EPICure study demonstrated persistent airflow limitation and increased respiratory morbidity among children born extremely preterm, with greater impairment in those with a history of BPD [11]. Similarly, longitudinal studies have shown less favorable lung-function trajectories in preterm-born children with BPD [19]. Nevertheless, the relationship between neonatal disease severity and later respiratory function is not uniform. Impaired airway function has also been reported in preterm-born children without BPD, and some studies have found that deterioration in airflow during childhood may occur independently of the presence of severe neonatal respiratory disease [19,20].

Several factors may explain the absence of significant associations in our cohort. First, the relatively small preterm cohort (*n* = 46), particularly the limited number of children in individual neonatal subgroups, may have reduced the statistical power to detect clinically relevant associations with factors such as BPD, mechanical ventilation, gestational age, and oxygen exposure. As the study was not prospectively powered to evaluate these secondary associations, the possibility of type II error cannot be excluded. Accordingly, these negative findings should be considered exploratory and should not be interpreted as evidence that later peripheral airway dysfunction is independent of neonatal disease severity. Second, lung development and alveolarization continue throughout childhood and adolescence, providing some potential for postnatal compensatory growth [21]. This ongoing development may partially attenuate the relationship between neonatal lung injury and pulmonary function measured several years later, although it should not be interpreted as complete recovery or normalization of the lungs. In addition, genetic susceptibility, childhood respiratory infections, environmental exposures, somatic growth, and variations in airway and parenchymal development may modify individual lung-function trajectories after preterm birth [12]. Consequently, peripheral airway dysfunction at school age may represent a heterogeneous developmental phenotype reflecting the cumulative effects of prematurity and subsequent life-course modifiers rather than the direct consequence of a single neonatal variable. Larger prospective cohorts with sufficient statistical power for neonatal subgroup analyses are needed to clarify these relationships.

Notably, no significant associations were detected between IOS parameters and atopic diseases, aeroallergen sensitization, total IgE levels, or peripheral eosinophil counts. Although these findings do not exclude a contribution from atopic or inflammatory mechanisms, they suggest that factors other than classical eosinophilic airway inflammation may contribute to the observed oscillometric abnormalities. Larger studies are needed to clarify the underlying mechanisms.

From a clinical perspective, our findings indicate that peripheral airway abnormalities can be detected in school-aged children born preterm, even in the absence of obvious respiratory symptoms. Although spirometry is widely used in long-term follow-up, it has limited ability to detect early mechanical changes in the peripheral airways. IOS, on the other hand, may be a valuable complementary method, particularly for the long-term follow-up of preterm children, as it can be performed during tidal breathing, is easy to use in children and provides complementary information on peripheral airway mechanics. The absence of a significant between-group difference in R20, despite higher R5 and R5–R20 values in the preterm group, is consistent with this interpretation. The long-term clinical significance of these peripheral airway abnormalities remains uncertain and should be evaluated in prospective longitudinal studies.

The strengths of our study include the assessment of IOS parameters in school-aged children born preterm using standardized oscillometric measurements with the Vyntus™ IOS system, the examination of detailed neonatal records, and the comparative analysis with a control group. However, this study has several limitations. The single-center, cross-sectional design precludes causal inference, and the relatively small sample size may have limited our ability to detect associations with certain neonatal variables. In addition, advanced pulmonary function assessments, such as bronchodilator response, lung volumes, and diffusion capacity, were not performed. The reference equations used for R5–R20 and AX were derived predominantly from a Mexican population and have not been specifically validated in Turkish children; therefore, some degree of reference-population bias cannot be excluded despite adjustment for major anthropometric determinants. Selection bias related to recruitment should also be considered. Of the 180 potentially eligible preterm children identified from neonatal records, only 46 were ultimately included, largely because many families could not be contacted several years after neonatal discharge. Families who remained reachable and were willing and able to attend the study visit may have differed systematically from nonparticipants, while the requirement for adequate cooperation with pulmonary function testing may have preferentially selected healthier or more developmentally capable children. Conversely, healthy term-born controls were recruited from children attending outpatient clinics for routine visits and from children of hospital staff, which may have resulted in a particularly healthy control group and potentially amplified the observed between-group differences. Residual confounding related to respiratory morbidity also remains possible because asthma, recurrent wheezing, inhaled corticosteroid use, and sensitization were not characterized comparably in both groups. Nevertheless, the associations remained significant in sensitivity analyses excluding preterm participants with asthma and/or recurrent wheezing. Collectively, these factors may limit the representativeness of the study population and the generalizability of the observed differences in respiratory function.

## 5. Conclusions

Children born very preterm exhibited differences in oscillometric measures of respiratory mechanics at school age compared with term-born controls, particularly in parameters reflecting peripheral airway mechanics. Although no significant associations between peripheral airway dysfunction and individual neonatal characteristics were detected, the limited sample size precludes firm conclusions regarding the contribution of these factors. These findings suggest that IOS may have potential as a complementary tool for assessing respiratory mechanics in children born preterm, including those without overt respiratory symptoms. However, given the cross-sectional design, the clinical and prognostic significance of these abnormalities remains uncertain. Prospective longitudinal studies are needed to determine whether serial IOS measurements can identify children at increased risk of subsequent respiratory morbidity and provide additional value in long-term respiratory follow-up.

## Figures and Tables

**Table 1:** Demographic and clinical characteristics of the preterm-born cohort (*n* = 46).

Category	Variable	*n* (%)
Demographics	Male sex	21 (45.7)
	Age, years, median (Q1-Q3)	7 (7–7.5)
	Consanguineous marriage	15 (32.6)
	Gestational week, median (Q1-Q3)	29 (28–32)
	Birth weight, kg, median (Q1-Q3)	1.25 (0.89–2.0)
	SGA	11 (23.9)
	History of chorioamnionitis	10 (21.7)
	Neonatal sepsis	30 (65.2)
	Necrotizing enterocolitis	2 (4.3)
	Neonatal PDA	9 (19.6)
	Neonatal IVH	10 (21.7)
	Neonatal premature apnea	20 (43.5)
	BPD	16 (34.8)
	Mechanical ventilation	19 (41.3)
	Mechanical ventilation, days, median (Q1-Q3)	1 (0–1)
	CPAP	40 (87)
	Oxygen therapy duration, days, median (Q1-Q3)	3.5 (1–19)
	Length of NICU stay (days), median (Q1-Q3)	43 (19–75)
	Surfactant administration	10 (21.7)
	History of postnatal steroids	8 (17.4)
	History of prenatal steroids	14 (30.4)
	History of inhaler steroids longer than 2 months	8 (17.4)
	Hospitalization due to pneumonia	5 (10.9)
	Intensive care unit admission due to pneumonia	2 (4.3)
	Breastfeeding > 6 months	19 (41.3)
	Exposure to smoking	10 (21.7)
	Pet ownership	3 (6.5)
Personal history of atopy	Asthma/recurrent wheezing	10 (21.7)
	Allergic rhinitis	7 (15.2)
	Eczema history	3 (6.5)
	Food allergy history	1 (2.2)
Family history of atopy	Asthma	6 (13)
	Allergic rhinitis	11 (23.9)
	Eczema history	6 (13)
Personal history of co-morbidity	Motor developmental problem	11 (23.9)
	Visual problem	14 (30.4)
	Hearing problem	3 (6.5)
	Special education report	6 (13)
	Physical therapy	8 (17.4)
Laboratory	Total IgE, IU/mL, median (Q1-Q3)	56 (15.3–148)
	Eosinophil count × 10^3^/mm^3^, median (Q1-Q3)	0.29 (0.1–0.84)
	Aeroallergen sensitization	12 (26)

BPD: Bronchopulmonary dysplasia, CPAP: Continuous positive airway pressure, IVH: Intraventricular hemorrhage, NICU: Neonatal intensive care unit, PDA: Patent ductus arteriosus, SGA: Small for gestational age.

**Table 2:** Demographic Characteristics and Oscillometric Measurements of the Preterm and Healthy Control Groups.

Variables	Preterm Group (*n* = 46)	Healthy Control Group (*n* = 40)	*p*-Value
Male sex, *n* (%)	21 (45.7)	20 (50)	0.85
Age, years, median (Q1-Q3)	7 (7–7.5)	7.5 (7–8)	0.072
Height z-score, median (Q1-Q3)	−0.24 (−1.2–0.68)	0.03 (−0.34–1.07)	0.016
Weight z-score, median (Q1-Q3)	−0.54 (−1.8–0.75)	0.09 (−0.19–0.71)	0.024
Smoking exposure, *n* (%)	10 (21.7)	13 (32.5)	0.37
R5 (kPa·s/L), median (Q1-Q3)	0.75 (0.56–0.83)	0.48 (0.43–0.70)	0.001
R20 (kPa·s/L), median (Q1-Q3)	0.36 (0.3–0.45)	0.33 (0.25–0.45)	0.459
R5–R20 (kPa·s/L), median (Q1-Q3)	0.37 (0.24–0.49)	0.19 (0.12–0.24)	0.001
X5 (kPa·s/L), median (Q1-Q3)	−0.15 [−0.22–(−0.01)]	−0.09 [−0.15–(−0.07)]	0.094
X20 (kPa·s/L), median (Q1-Q3)	−0.07 [−0.14–(−0.03)]	−0.05 [−0.1–(−0.01)]	0.08
AX (kPa/L), median (Q1-Q3)	2.93 (1.98–3.82)	1.41 (1.11–2.74)	0.001
Fres (Hz), median (Q1-Q3)	22.01 (16.5–25.1)	22.2 (20.2–25.9)	0.145

**Table 3:** Oscillometric Measurements, Abnormality Criteria, and Frequency of Abnormal IOS Findings in children born prematurely (*n* = 46).

Variables	Impulse Oscillometry Parameters	Abnormality Criterion (ULN/LLN z Score)	Abnormal Results, *n* (%)
R5 (kPa·s/L), median (Q1-Q3)	0.75 (0.56–0.83)	z > +1.645	12 (26.1)
R20 (kPa·s/L), median (Q1-Q3)	0.36 (0.3–0.45)	z > +1.645	9 (19.6)
R5–R20 (kPa·s/L), median (Q1-Q3)	0.37 (0.24–0.49)	z > +1.645	16 (34.8)
X5 (kPa·s/L), median (Q1-Q3)	−0.15 [−0.22–(−0.01)]	z < −1.645	8 (17.4)
X20 (kPa·s/L), median (Q1-Q3)	−0.07 [−0.14–(−0.03)]	z < −1.645	9 (19.6)
AX (kPa/L), median (Q1-Q3)	2.93 (1.98–3.82)	z > +1.645	10 (21.7)
Fres (Hz), median (Q1-Q3)	22.01 (16.5–25.1)	z > +1.645	9 (19.6)

**Table 4:** Multivariable linear regression analyses of IOS parameters according to prematurity status and anthropometric measures.

	Outcome/Predictor	B	SE	Standardized β	95% CI for B	*p* Value
**R5**	Prematurity status	**0.149**	**0.052**	**0.329**	**0.047 to 0.252**	**0.005**
	Age	0.037	0.041	0.096	−0.046 to 0.119	0.379
	Height z-score	−0.024	0.027	−0.114	−0.078 to 0.030	0.376
	Weight z-score	0.013	0.021	0.074	−0.030 to 0.055	0.556
**R5-R20**	Prematurity status	**0.181**	**0.040**	**0.474**	**0.101 to 0.260**	**<0.001**
	Age	0.005	0.032	0.015	−0.059 to 0.069	0.880
	Height z-score	−0.017	0.021	−0.093	−0.058 to 0.025	0.434
	Weight z-score	0.002	0.016	0.014	−0.031 to 0.035	0.907
**AX**	Prematurity status	**1.007**	**0.309**	**0.370**	**0.393 to 1.621**	**0.002**
	Age	0.412	0.248	0.181	−0.082 to 0.906	0.101
	Height z-score	0.033	0.162	0.026	−0.289 to 0.356	0.837
	Weight z-score	0.075	0.127	0.074	−0.178 to 0.328	0.558

Abbreviations: B, unstandardized regression coefficient; SE, standard error; β, standardized regression coefficient; CI, confidence interval; R5, resistance at 5 Hz; R20, resistance at 20 Hz; R5–R20, frequency-dependent difference in resistance; AX, area of reactance. Prematurity status was coded as 0 = term-born control and 1 = preterm. Age, height z-score, and weight z-score were entered as continuous variables. All predictors were entered simultaneously. Bold values indicate *p* < 0.05. Model fit: R5: R^2^ = 0.122, adjusted R^2^ = 0.079, F (4,81) = 2.824, *p* = 0.030; R5–R20: R^2^ = 0.251, adjusted R^2^ = 0.214, F (4,81) = 6.772, *p* < 0.001; AX: R^2^ = 0.125, adjusted R^2^ = 0.082, F (4,81) = 2.889, *p* = 0.027. All models included 86 participants.

## Data Availability

The data presented in this study are available on request from the corresponding author.

## Supplementary Materials

The following supporting information can be downloaded at: https://www.mdpi.com/article/10.3390/jcm15176692/s1, Table S1. Univariable associations of perinatal, neonatal, clinical, and environmental characteristics with R5–R20 among children born preterm.
