# Supplementary material for: Peripheral Airway Dysfunction Assessed by Impulse Oscillometry in School-Aged Children Born Preterm: A Cross-Sectional Comparative Study
Source: J Clin Med. 2026 Aug 28;15(17):6692. doi: 10.3390/jcm15176692 (PMC13566541; doi:10.3390/jcm15176692)
Supplement: Supplementary file 1 [file jcm-15-06692-s001.zip › jcm-4487257-supplementary.pdf]

Supplementary Table S1. Univariable associations of perinatal, neonatal, clinical, and environmental characteristics with R5–R20 among children born preterm

Predictor	Summary*	B	SE	β	95% CI for B	p value
**Continuous perinatal/neonatal characteristics**						
Gestational age, weeks, median (Q1-Q3)	29 (28-32)	0.014	0.013	0.160	− 0.012 to 0.040	0.288
Birth weight, g†, median (Q1-Q3)	1.25 (0.89-2.0)	**0.021**	0.013	0.235	− 0.005 to 0.047	**0.116**
NICU length of stay, days, median (Q1-Q3)	43 (19-75)	− 0.001	0.001	− 0.145	− 0.002 to 0.001	0.335
Mechanical ventilation duration, days, median (Q1-Q3)	1 (0-1)	− 0.014	0.010	− 0.205	− 0.034 to 0.006	0.171
Oxygen therapy duration, days, median (Q1-Q3)	3.5 (1-19)	− 0.002	0.002	− 0.171	− 0.006 to 0.002	0.256
**Categorical perinatal/neonatal characteristics**						
Bronchopulmonary dysplasia, n (%)	R5–R20 (+) group:5 (31.3)<br>R5–R20 (-) group:9 (30)	− 0.076	0.065	− 0.173	− 0.208 to 0.056	0.251
Mechanical ventilation, n (%)	R5–R20 (+) group:8 (50)<br>R5–R20 (-) group:11 (36.7)	0.057	0.061	0.139	− 0.067 to 0.181	0.357
Surfactant administration, n (%)	R5–R20 (+) group:3 (18.8)<br>R5–R20 (-) group:7 (23.3)	− 0.130	0.071	− 0.264	− 0.273 to 0.014	0.077
Neonatal sepsis, n (%)	R5–R20 (+) group:12 (75)<br>R5–R20 (-) group:18 (60)	0.030	0.064	0.070	− 0.099 to 0.159	0.645
Necrotizing enterocolitis, n (%)	R5–R20 (+) group:1 ((6.3)<br>R5–R20 (-) group:1 (3.3)	− 0.120	0.149	− 0.120	− 0.419 to 0.180	0.426
Patent ductus arteriosus, n (%)	R5–R20 (+) group:3 (18.8)<br>R5–R20 (-) group:6 (20)	− 0.097	0.076	− 0.190	− 0.250 to 0.055	0.205
Intraventricular hemorrhage, n (%)	R5–R20 (+) group:4 (25)<br>R5–R20 (-) group:6 (20)	− 0.025	0.074	− 0.050	− 0.174 to 0.124	0.740
Apnea of prematurity, n (%)	R5–R20 (+) group:8 (50)<br>R5–R20 (-) group:12 (40)	− 0.031	0.061	− 0.075	− 0.155 to 0.093	0.620
**Post-neonatal clinical characteristics**						
Lower respiratory tract infection requiring hospitalization, n (%)	R5–R20 (+) group:2 (12.5)<br>R5–R20 (-) group:3 (10)	− 0.096	0.097	− 0.147	− 0.291 to 0.100	0.329
Hearing impairment, n (%)	R5–R20 (+) group:1 (6.3)<br>R5–R20 (-) group:2 (6.7)	0.015	0.124	0.018	− 0.234 to 0.264	0.904
Visual disorders, n (%)	R5–R20 (+) group: 0 (0)<br>R5–R20 (-) group:8 (26.7)	− 0.104	0.079	− 0.194	− 0.263 to 0.056	0.197
Motor developmental delay, n (%)	R5–R20 (+) group:5 (31.3)<br>R5–R20 (-) group:6 (20)	0.053	0.071	0.112	− 0.090 to 0.197	0.457
Special education requirement, n (%)	R5–R20 (+) group: 3 (18.8)<br>R5–R20 (-) group:3 (10)	0.097	0.090	0.161	− 0.084 to 0.277	0.287
**Allergic/environmental characteristics**						
Aeroallergen sensitization, n (%)	R5–R20 (+) group:4 (25)<br>R5–R20 (-) group:3 (10)	0.064	0.106	0.122	− 0.155 to 0.282	0.553
Smoking Exposure, n (%)	R5–R20 (+) group: 3 (18.8)<br>R5–R20 (-) group:7 (23.3)	0.061	0.174	0.053	− 0.289 to 0.411	0.727
Pet ownership, n (%)	R5–R20 (+) group:2 (12.5)<br>R5–R20 (-) group: 1 (3.3)	0.004	0.124	0.005	− 0.245 to 0.254	0.973

Table footnote: B, unstandardized regression coefficient; SE, standard error; β, standardized regression coefficient; CI, confidence interval; NICU, neonatal intensive care unit; R5–R20, frequency-dependent difference in resistance between 5 and 20 Hz. Each predictor was examined separately in a univariable linear regression model with R5–R20 as the dependent variable. Analyses were restricted to children born preterm. For continuous predictors, B represents the estimated change in R5–R20 per one-unit increase in the predictor. Binary variables were coded as 0=no and 1=yes; therefore, B represents the estimated mean difference in R5–R20 for participants with versus without the specified characteristic.

* **Summary:** Continuous variables are presented as median (Q1–Q3) for the overall preterm cohort. For categorical variables, n and median R5–R20 (Q1–Q3) are presented separately for the (+) and (−) groups. Of the 46 children born preterm, 16 (34.8%) had R5–R20 values above the upper limit of normal.

† **Birth weight:** I would change this row to “Birth weight, per 100 g” after rerunning the regression with birth weight divided by 100. This will give a readable B and 95% CI rather than SPSS's rounded 0.000–0.000.
